# 1,2-Diphenyl-1*H*-benzimidazole

**DOI:** 10.1107/S1600536812044960

**Published:** 2012-11-03

**Authors:** S. Rosepriya, A. Thiruvalluvar, K. Jayamoorthy, J. Jayabharathi, Sema Öztürk Yildirim, R. J. Butcher

**Affiliations:** a Postgraduate Research Department of Physics, Rajah Serfoji Government College (Autonomous), Thanjavur 613 005, Tamilnadu, India; b Department of Chemistry, Annamalai University, Annamalai Nagar 608 002, Tamilnadu, India; c Department of Chemistry, Howard University, 525 College Street NW, Washington, DC 20059, USA; d Department of Physics, Faculty of Sciences, Erciyes University, 38039 Kayseri, Turkey

## Abstract

In the title mol­ecule, C_19_H_14_N_2_, the benzimidazole unit is close to being planar [maximum deviation = 0.0102 (6) Å] and forms dihedral angles of 55.80 (2) and 40.67 (3)° with the adjacent phenyl rings; the dihedral angle between the phenyl rings is 62.37 (3)°. In the crystal, one C—H⋯N hydrogen bond and three weak C—H⋯π inter­actions involving the fused benzene ring and the imidazole ring are observed, leading to a three-dimensional architecture.

## Related literature

For the use of benzoimidazoles and phenanthroimidazoles as light-emitting devices and dye-sensitized solar cells, see: Fang *et al.* (2007 ▶); Ge *et al.* (2008 ▶); Lai *et al.* (2008 ▶); Shin *et al.* (2007 ▶); Tsai *et al.* (2007 ▶). For a closely related crystal structure, see: Jayamoorthy *et al.* (2012 ▶).
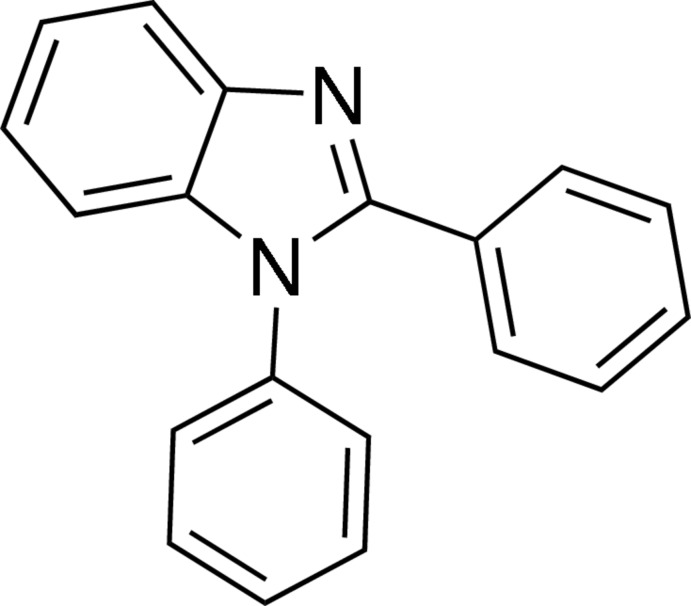


## Experimental

### 

#### Crystal data

C_19_H_14_N_2_
*M* *_r_* = 270.32
Monoclinic, 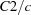

*a* = 10.1878 (3) Å
*b* = 16.6399 (4) Å
*c* = 17.4959 (5) Å
β = 106.205 (3)°
*V* = 2848.13 (14) Å^3^
*Z* = 8
Mo *K*α radiation
μ = 0.08 mm^−1^
*T* = 123 K
0.60 × 0.40 × 0.35 mm

#### Data collection

Agilent Xcalibur Ruby Gemini diffractometer
Absorption correction: multi-scan (*CrysAlis PRO*; Agilent, 2012 ▶) *T*_min_ = 0.957, *T*_max_ = 0.974
25253 measured reflections
7296 independent reflections
5803 reflections with *I* > 2σ(*I*)
*R*_int_ = 0.028

#### Refinement

*R*[*F* ^2^ > 2σ(*F* ^2^)] = 0.052
*wR*(*F* ^2^) = 0.137
*S* = 1.06
7296 reflections
190 parameters
H-atom parameters constrained
Δρ_max_ = 0.52 e Å^−3^
Δρ_min_ = −0.25 e Å^−3^

### 

Data collection: *CrysAlis PRO* (Agilent, 2012 ▶); cell refinement: *CrysAlis PRO*; data reduction: *CrysAlis PRO*; program(s) used to solve structure: *SHELXS97* (Sheldrick, 2008 ▶); program(s) used to refine structure: *SHELXL97* (Sheldrick, 2008 ▶); molecular graphics: *ORTEP-3* (Farrugia, 1997 ▶) and *PLATON* (Spek, 2009 ▶); software used to prepare material for publication: *PLATON*.

## Supplementary Material

Crystal structure: contains datablock(s) global, I. DOI: 10.1107/S1600536812044960/hg5265sup1.cif

Structure factors: contains datablock(s) I. DOI: 10.1107/S1600536812044960/hg5265Isup2.hkl

Supplementary material file. DOI: 10.1107/S1600536812044960/hg5265Isup3.cdx

Supplementary material file. DOI: 10.1107/S1600536812044960/hg5265Isup4.cml

Additional supplementary materials:  crystallographic information; 3D view; checkCIF report

## Figures and Tables

**Table 1:** Hydrogen-bond geometry (Å, °) *Cg*2 is the centroid of the C4–C9 fused benzene ring and *Cg*1 is the centroid of the N1/C2/N3/C9/C8 imidazole ring.

*D*—H⋯*A*	*D*—H	H⋯*A*	*D*⋯*A*	*D*—H⋯*A*
C14—H14⋯N3^i^	0.93	2.62	3.4829 (11)	154
C16—H16⋯*Cg*2^ii^	0.93	2.68	3.4843 (9)	146
C22—H22⋯*Cg*1^iii^	0.93	2.91	3.3966 (9)	114
C23—H23⋯*Cg*2^iii^	0.93	2.83	3.4609 (9)	126

Symmetry codes: (i) 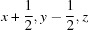; (ii) 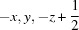; (iii) 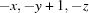.

**Fig. 1.**
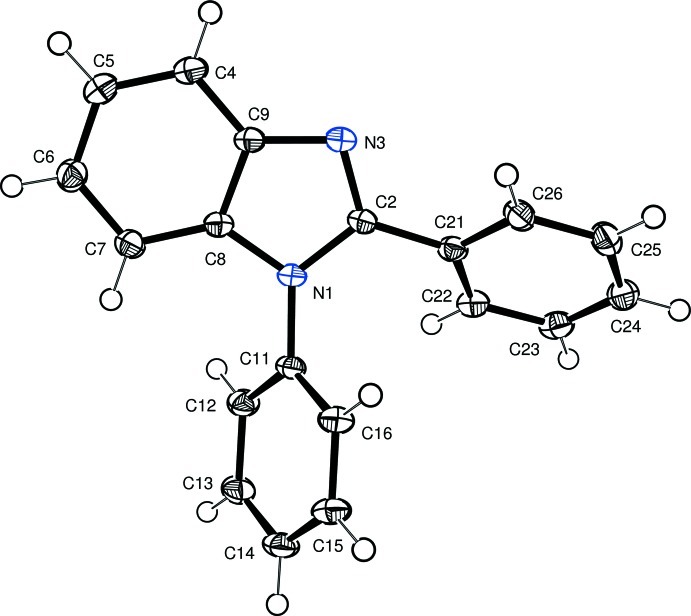
The molecular structure of the title compound, with displacement ellipsoids drawn at the 50% probability level. H atoms are shown as small spheres of arbitrary radius.

**Fig. 2.**
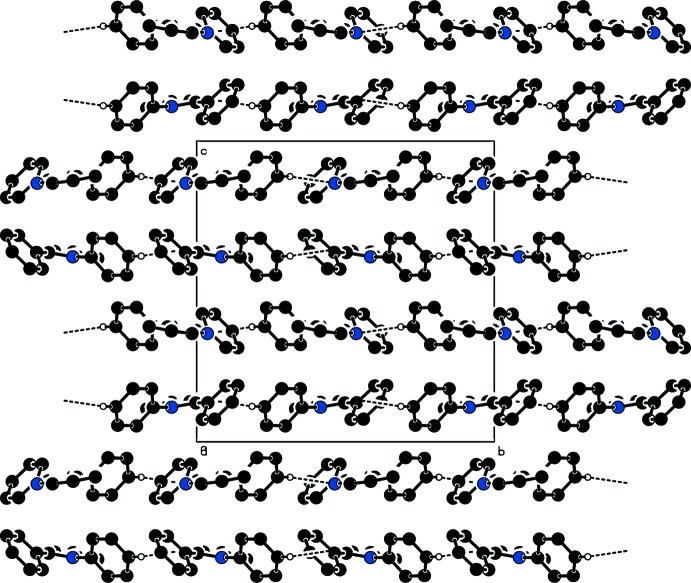
The packing of the title compound, viewed down the a axis. Dashed lines indicate hydrogen bonds. H atoms not involved in hydrogen bonding have been omitted.

## supplementary crystallographic information

### Comment

Fused imidazole derivatives such as benzoimidazoles and phenanthroimidazoles [Fang *et al.*, (2007), Ge *et al.*, (2008), Lai *et al.*, (2008)] have been used in the fabrication of light-emitting devices, employing them as electron-transporting layer and as sensitizers in dye-sensitized solar cells [Shin *et al.*, (2007), Tsai *et al.*, (2007)] due to their wide optical absorption, bright luminescence and bipolar transport characteristics. Since our research group is working in organic light emitting devices, we are interested to use the title compound as ligand for synthesizing Ir(III) complexes. Jayamoorthy *et al.*, (2012) have reported a closely related crystal structure of 2-(4-Fluorophenyl)-1-phenyl-1*H*-benzimidazole.

In the title molecule, C_19_H_14_N_2_ (Fig. 1), the benzimidazole unit is almost planar [maximum deviation = 0.0102 (6) Å for C2]. The dihedral angles between the planes of the benzimidazole and the phenyl ring at N1 and the phenyl at C2 are 55.80 (2) and 40.67 (3)°, respectively. The dihedral angle between the planes of the adjacent phenyl rings is 62.37 (3)°. Intermolecular C14—H14···N3 hydrogen bond and weak C16—H16···π interaction involving the fused benzene ring, C22—H22···π interaction involving the imidazole ring and C23—H23···π interaction involving the fused benzene ring are found in the crystal structure (Fig. 2, Table 1).

### Experimental

The pure *N*-phenyl-*o*-phenylenediamine (3.128 g, 17 mmol) in ethanol (10 ml), benzaldehyde (1.72 ml, 17 mmol) and ammonium acetate (3 g) was added about 1 h by maintaining the temperature at 353 K. The reaction mixture was refluxed for appropriate time and the completion of reaction was monitored by TLC, finally the reaction extracted with dichloromethane. The solid separated was purified by column chromatography using petroleum ether: ethyl acetate as the eluent. Yield: 2.49 g (50%). The title compound was dissolved in acetonitrile and allowed to slow evaporation for two days to obtain crystals suitable for X-ray diffraction studies.

### Refinement

The H atoms were positioned geometrically and allowed to ride on their parent atoms, with C—H = 0.93 Å. *U*_iso_(H) = *1.2U*_eq_(C).

### Crystal data

**Table:**

C_19_H_14_N_2_	*F*(000) = 1136
*M**_r_* = 270.32	*D*_x_ = 1.261 Mg m^−^^3^
Monoclinic, *C*2/*c*	Melting point: 380 K
Hall symbol: -C 2yc	Mo *K*α radiation, λ = 0.71073 Å
*a* = 10.1878 (3) Å	Cell parameters from 6755 reflections
*b* = 16.6399 (4) Å	θ = 3.4–37.7°
*c* = 17.4959 (5) Å	µ = 0.08 mm^−^^1^
β = 106.205 (3)°	*T* = 123 K
*V* = 2848.13 (14) Å^3^	Block, colourless
*Z* = 8	0.60 × 0.40 × 0.35 mm

### Data collection

**Table:**

Agilent Xcalibur Ruby Gemini diffractometer	7296 independent reflections
Radiation source: Enhance (Mo) X-ray Source	5803 reflections with *I* > 2σ(*I*)
Graphite monochromator	*R*_int_ = 0.028
Detector resolution: 10.5081 pixels mm^-1^	θ_max_ = 37.8°, θ_min_ = 3.5°
ω scans	*h* = −17→16
Absorption correction: multi-scan (*CrysAlis PRO*; Agilent, 2012)	*k* = −27→27
*T*_min_ = 0.957, *T*_max_ = 0.974	*l* = −29→29
25253 measured reflections	

### Refinement

**Table:**

Refinement on *F*^2^	Primary atom site location: structure-invariant direct methods
Least-squares matrix: full	Secondary atom site location: difference Fourier map
*R*[*F*^2^ > 2σ(*F*^2^)] = 0.052	Hydrogen site location: inferred from neighbouring sites
*wR*(*F*^2^) = 0.137	H-atom parameters constrained
*S* = 1.06	*w* = 1/[σ^2^(*F*_o_^2^) + (0.063*P*)^2^ + 1.1529*P*] where *P* = (*F*_o_^2^ + 2*F*_c_^2^)/3
7296 reflections	(Δ/σ)_max_ = 0.001
190 parameters	Δρ_max_ = 0.52 e Å^−^^3^
0 restraints	Δρ_min_ = −0.25 e Å^−^^3^

### Special details

**Table:**

Geometry. Bond distances, angles *etc*. have been calculated using the rounded fractional coordinates. All su's are estimated from the variances of the (full) variance-covariance matrix. The cell e.s.d.'s are taken into account in the estimation of distances, angles and torsion angles
Refinement. Refinement of *F*^2^ against ALL reflections. The weighted *R*-factor *wR* and goodness of fit *S* are based on *F*^2^, conventional *R*-factors *R* are based on *F*, with *F* set to zero for negative *F*^2^. The threshold expression of *F*^2^ > 2σ(*F*^2^) is used only for calculating *R*-factors(gt) *etc*. and is not relevant to the choice of reflections for refinement. *R*-factors based on *F*^2^ are statistically about twice as large as those based on *F*, and *R*- factors based on ALL data will be even larger.

### Fractional atomic coordinates and isotropic or equivalent isotropic displacement parameters (Å^2^)

**Table:**

	*x*	*y*	*z*	*U*_iso_*/*U*_eq_	
N1	−0.02193 (7)	0.41603 (4)	0.11513 (4)	0.0171 (2)	
N3	−0.10490 (7)	0.53588 (4)	0.13995 (4)	0.0203 (2)	
C2	0.00172 (8)	0.49743 (5)	0.12929 (4)	0.0175 (2)	
C4	−0.33775 (9)	0.48436 (6)	0.13998 (5)	0.0236 (2)	
C5	−0.41655 (9)	0.41533 (6)	0.13157 (6)	0.0246 (2)	
C6	−0.36456 (9)	0.34053 (6)	0.11689 (5)	0.0230 (2)	
C7	−0.23322 (9)	0.33246 (5)	0.10945 (5)	0.0204 (2)	
C8	−0.15513 (8)	0.40216 (5)	0.11787 (4)	0.0175 (2)	
C9	−0.20463 (8)	0.47749 (5)	0.13339 (5)	0.0189 (2)	
C11	0.07472 (8)	0.35411 (5)	0.11440 (4)	0.0170 (2)	
C12	0.04519 (9)	0.29794 (5)	0.05292 (5)	0.0219 (2)	
C13	0.13562 (10)	0.23476 (5)	0.05505 (6)	0.0249 (2)	
C14	0.25548 (10)	0.22842 (5)	0.11667 (6)	0.0249 (2)	
C15	0.28460 (9)	0.28592 (6)	0.17671 (5)	0.0244 (2)	
C16	0.19401 (9)	0.34852 (5)	0.17652 (5)	0.0207 (2)	
C21	0.13044 (8)	0.53770 (5)	0.12998 (5)	0.0180 (2)	
C22	0.20146 (9)	0.51883 (5)	0.07441 (5)	0.0206 (2)	
C23	0.32060 (9)	0.56028 (6)	0.07548 (5)	0.0235 (2)	
C24	0.36977 (9)	0.61969 (6)	0.13203 (6)	0.0253 (2)	
C25	0.29922 (10)	0.63848 (5)	0.18729 (6)	0.0254 (2)	
C26	0.17964 (9)	0.59817 (5)	0.18609 (5)	0.0217 (2)	
H4	−0.37244	0.53382	0.14972	0.0283*	
H5	−0.50543	0.41859	0.13571	0.0296*	
H6	−0.41968	0.29520	0.11203	0.0275*	
H7	−0.19908	0.28296	0.09934	0.0245*	
H12	−0.03397	0.30262	0.01096	0.0262*	
H13	0.11560	0.19638	0.01481	0.0299*	
H14	0.31565	0.18613	0.11775	0.0299*	
H15	0.36562	0.28249	0.21748	0.0293*	
H16	0.21306	0.38614	0.21746	0.0248*	
H22	0.16910	0.47870	0.03688	0.0248*	
H23	0.36739	0.54820	0.03826	0.0281*	
H24	0.44992	0.64692	0.13297	0.0303*	
H25	0.33243	0.67821	0.22513	0.0305*	
H26	0.13210	0.61136	0.22266	0.0260*	

### Atomic displacement parameters (Å^2^)

**Table:**

	*U*^11^	*U*^22^	*U*^33^	*U*^12^	*U*^13^	*U*^23^
N1	0.0164 (3)	0.0143 (3)	0.0209 (3)	0.0033 (2)	0.0056 (2)	−0.0001 (2)
N3	0.0191 (3)	0.0166 (3)	0.0259 (3)	0.0036 (2)	0.0075 (2)	−0.0006 (2)
C2	0.0188 (3)	0.0147 (3)	0.0193 (3)	0.0029 (2)	0.0057 (2)	0.0005 (2)
C4	0.0185 (3)	0.0227 (4)	0.0298 (4)	0.0047 (3)	0.0073 (3)	−0.0014 (3)
C5	0.0175 (3)	0.0274 (4)	0.0289 (4)	0.0022 (3)	0.0063 (3)	0.0002 (3)
C6	0.0199 (3)	0.0235 (4)	0.0242 (3)	−0.0013 (3)	0.0041 (3)	0.0005 (3)
C7	0.0207 (3)	0.0175 (3)	0.0224 (3)	0.0011 (3)	0.0049 (3)	−0.0001 (3)
C8	0.0168 (3)	0.0169 (3)	0.0185 (3)	0.0031 (2)	0.0043 (2)	0.0006 (2)
C9	0.0177 (3)	0.0171 (3)	0.0220 (3)	0.0035 (2)	0.0055 (2)	−0.0002 (2)
C11	0.0183 (3)	0.0143 (3)	0.0194 (3)	0.0039 (2)	0.0069 (2)	0.0012 (2)
C12	0.0214 (3)	0.0205 (3)	0.0236 (3)	0.0028 (3)	0.0062 (3)	−0.0045 (3)
C13	0.0275 (4)	0.0197 (4)	0.0304 (4)	0.0034 (3)	0.0130 (3)	−0.0051 (3)
C14	0.0271 (4)	0.0198 (3)	0.0319 (4)	0.0088 (3)	0.0148 (3)	0.0038 (3)
C15	0.0227 (4)	0.0248 (4)	0.0256 (4)	0.0095 (3)	0.0064 (3)	0.0054 (3)
C16	0.0222 (3)	0.0197 (3)	0.0196 (3)	0.0058 (3)	0.0049 (3)	0.0012 (3)
C21	0.0190 (3)	0.0157 (3)	0.0200 (3)	0.0033 (2)	0.0066 (2)	0.0029 (2)
C22	0.0212 (3)	0.0214 (3)	0.0199 (3)	0.0043 (3)	0.0068 (3)	0.0020 (3)
C23	0.0226 (4)	0.0250 (4)	0.0253 (4)	0.0058 (3)	0.0107 (3)	0.0063 (3)
C24	0.0225 (4)	0.0217 (4)	0.0335 (4)	0.0009 (3)	0.0109 (3)	0.0060 (3)
C25	0.0275 (4)	0.0181 (3)	0.0322 (4)	−0.0028 (3)	0.0109 (3)	−0.0008 (3)
C26	0.0253 (4)	0.0166 (3)	0.0255 (3)	−0.0001 (3)	0.0109 (3)	−0.0007 (3)

### Geometric parameters (Å, º)

**Table:**

N1—C2	1.3859 (11)	C21—C26	1.3974 (12)
N1—C8	1.3905 (11)	C22—C23	1.3916 (13)
N1—C11	1.4277 (11)	C23—C24	1.3894 (14)
N3—C2	1.3180 (11)	C24—C25	1.3918 (14)
N3—C9	1.3872 (11)	C25—C26	1.3857 (14)
C2—C21	1.4697 (12)	C4—H4	0.9300
C4—C5	1.3855 (14)	C5—H5	0.9300
C4—C9	1.3975 (13)	C6—H6	0.9300
C5—C6	1.4042 (14)	C7—H7	0.9300
C6—C7	1.3866 (13)	C12—H12	0.9300
C7—C8	1.3908 (12)	C13—H13	0.9300
C8—C9	1.4056 (12)	C14—H14	0.9300
C11—C12	1.3930 (11)	C15—H15	0.9300
C11—C16	1.3891 (12)	C16—H16	0.9300
C12—C13	1.3915 (13)	C22—H22	0.9300
C13—C14	1.3892 (15)	C23—H23	0.9300
C14—C15	1.3903 (13)	C24—H24	0.9300
C15—C16	1.3911 (13)	C25—H25	0.9300
C21—C22	1.4000 (12)	C26—H26	0.9300
			
C2—N1—C8	106.18 (7)	C24—C25—C26	120.20 (9)
C2—N1—C11	128.39 (7)	C21—C26—C25	120.04 (8)
C8—N1—C11	124.24 (7)	C5—C4—H4	121.00
C2—N3—C9	105.18 (7)	C9—C4—H4	121.00
N1—C2—N3	113.00 (7)	C4—C5—H5	119.00
N1—C2—C21	123.87 (7)	C6—C5—H5	119.00
N3—C2—C21	123.11 (7)	C5—C6—H6	119.00
C5—C4—C9	118.06 (9)	C7—C6—H6	119.00
C4—C5—C6	121.16 (9)	C6—C7—H7	122.00
C5—C6—C7	121.75 (9)	C8—C7—H7	122.00
C6—C7—C8	116.60 (8)	C11—C12—H12	120.00
N1—C8—C7	132.04 (8)	C13—C12—H12	120.00
N1—C8—C9	105.36 (7)	C12—C13—H13	120.00
C7—C8—C9	122.59 (8)	C14—C13—H13	120.00
N3—C9—C4	129.88 (8)	C13—C14—H14	120.00
N3—C9—C8	110.28 (7)	C15—C14—H14	120.00
C4—C9—C8	119.85 (8)	C14—C15—H15	120.00
N1—C11—C12	119.36 (7)	C16—C15—H15	120.00
N1—C11—C16	119.76 (7)	C11—C16—H16	120.00
C12—C11—C16	120.83 (8)	C15—C16—H16	120.00
C11—C12—C13	119.18 (8)	C21—C22—H22	120.00
C12—C13—C14	120.68 (8)	C23—C22—H22	120.00
C13—C14—C15	119.35 (9)	C22—C23—H23	120.00
C14—C15—C16	120.80 (8)	C24—C23—H23	120.00
C11—C16—C15	119.14 (8)	C23—C24—H24	120.00
C2—C21—C22	121.66 (7)	C25—C24—H24	120.00
C2—C21—C26	118.58 (8)	C24—C25—H25	120.00
C22—C21—C26	119.72 (8)	C26—C25—H25	120.00
C21—C22—C23	119.83 (8)	C21—C26—H26	120.00
C22—C23—C24	120.13 (8)	C25—C26—H26	120.00
C23—C24—C25	120.07 (9)		
			
C8—N1—C2—N3	−0.56 (8)	C5—C6—C7—C8	0.47 (13)
C8—N1—C2—C21	−178.75 (7)	C6—C7—C8—N1	179.10 (8)
C11—N1—C2—N3	−168.36 (7)	C6—C7—C8—C9	0.20 (12)
C11—N1—C2—C21	13.45 (12)	N1—C8—C9—N3	0.02 (8)
C2—N1—C8—C7	−178.75 (8)	N1—C8—C9—C4	−179.91 (7)
C2—N1—C8—C9	0.30 (8)	C7—C8—C9—N3	179.18 (7)
C11—N1—C8—C7	−10.30 (13)	C7—C8—C9—C4	−0.76 (12)
C11—N1—C8—C9	168.74 (7)	N1—C11—C12—C13	−175.99 (8)
C2—N1—C11—C12	−134.48 (8)	C16—C11—C12—C13	1.25 (13)
C2—N1—C11—C16	48.25 (11)	N1—C11—C16—C15	177.36 (8)
C8—N1—C11—C12	59.73 (10)	C12—C11—C16—C15	0.14 (13)
C8—N1—C11—C16	−117.54 (9)	C11—C12—C13—C14	−1.38 (14)
C9—N3—C2—N1	0.56 (9)	C12—C13—C14—C15	0.13 (15)
C9—N3—C2—C21	178.77 (7)	C13—C14—C15—C16	1.29 (14)
C2—N3—C9—C4	179.58 (9)	C14—C15—C16—C11	−1.42 (14)
C2—N3—C9—C8	−0.35 (9)	C2—C21—C22—C23	177.85 (8)
N1—C2—C21—C22	40.41 (12)	C26—C21—C22—C23	0.10 (12)
N1—C2—C21—C26	−141.81 (8)	C2—C21—C26—C25	−178.67 (8)
N3—C2—C21—C22	−137.60 (8)	C22—C21—C26—C25	−0.85 (13)
N3—C2—C21—C26	40.18 (11)	C21—C22—C23—C24	0.64 (14)
C9—C4—C5—C6	0.03 (14)	C22—C23—C24—C25	−0.65 (14)
C5—C4—C9—N3	−179.31 (9)	C23—C24—C25—C26	−0.11 (14)
C5—C4—C9—C8	0.62 (12)	C24—C25—C26—C21	0.86 (14)
C4—C5—C6—C7	−0.60 (14)		

### Hydrogen-bond geometry (Å, º)

Cg2 is the centroid of the C4–C9 fused benzene ring and Cg1 is the centroid of the N1/C2/N3/C9/C8 imidazole ring.

**Table:**

*D*—H···*A*	*D*—H	H···*A*	*D*···*A*	*D*—H···*A*
C14—H14···N3^i^	0.93	2.62	3.4829 (11)	154
C16—H16···*Cg*2^ii^	0.93	2.68	3.4843 (9)	146
C22—H22···*Cg*1^iii^	0.93	2.91	3.3966 (9)	114
C23—H23···*Cg*2^iii^	0.93	2.83	3.4609 (9)	126

Symmetry codes: (i) *x*+1/2, *y*−1/2, *z*; (ii) −*x*, *y*, −*z*+1/2; (iii) −*x*, −*y*+1, −*z*.
